# Whole Genome Sequencing Provides New Insights Into the Genetic Diversity and Coat Color of Asiatic Wild Ass and Its Hybrids

**DOI:** 10.3389/fgene.2022.818420

**Published:** 2022-05-12

**Authors:** Hong Dong, Zheng Dong, Fuwen Wang, Gang Wang, Xiaoyu Luo, Chuzhao Lei, Jingbo Chen

**Affiliations:** ^1^ College of Animal Science and Technology, SHIHEZI University, Shihezi, China; ^2^ Key Laboratory of Animal Genetics, Breeding and Reproduction of Shaanxi Province, College of Animal Science and Technology, Northwest A&F University, Yangling, China

**Keywords:** asiatic wild ass, whole genome analysis, KITLG, genetic diversity, coat color

## Abstract

The diversity of livestock coat color results from human positive selection and is an indispensable part of breed registration. As an important biodiversity resource, Asiatic wild ass has many special characteristics, including the most visualized feature, its yellowish-brown coat color, and excellent adaptation. To explore the genetic mechanisms of phenotypic characteristics in Asiatic wild ass and its hybrids, we resequenced the whole genome of one Mongolian Kulan (a subspecies of Asiatic wild ass) and 29 Kulan hybrids (Mongolian Kulan ♂×Xinjiang♀), and the ancestor composition indicated the true lineage of the hybrids. XP-EHH (Cross Population Extended Haplotype Homozygosity), θπ-ratio (Nucleotide Diversity Ratio), CLR (Composite Likelihood Ratio) and θπ (Nucleotide Diversity) methods were used to detect the candidate regions of positive selection in Asiatic wild ass and its hybrids. Several immune genes (*DEFA1*, *DEFA5*, *DEFA7*, *GIMAP4*, *GIMAP1*, *IGLC1*, *IGLL5*, *GZMB* and *HLA*) were observed by the CLR and θπ methods. XP-EHH and θπ-ratio revealed that these genes are potentially responsible for coat color (*KITLG*) and meat quality traits (*PDE1B* and *MYLK2*). Furthermore, the heatmap was able to show the clear difference in the haplotype of the *KITLG* gene between the Kulan hybrids and Asiatic wild ass group and the Guanzhong black donkey group, which is a powerful demonstration of the key role of *KITLG* in donkey color. Therefore, our study may provide new insights into the genetic basis of coat color, meat quality traits and immunity of Asiatic wild ass and its hybrids.

## Introduction

Coat color is one of the most visualized breed features of livestock. Long-term natural selection allows animals to have a variety of coat colors, which has become the focus of animal breeding research. *KIT* gene plays a vital role in the occurrence of animal coat color, and it is the pigmentary switch in domestic animal species (Klungland and Vage, 2003). The diversiform coat color phenotypes segregating in Duroc hybrid pigs demonstrated that *KIT* is responsible for the complex variation (Wu et al., 2019). In addition, the coat color phenotypes of several species showed an indispensable association with the *KIT* gene (Zhang et al., 2015; Anello et al., 2019; Voß et al., 2020; Wen et al., 2021). The *KITLG* gene encodes the KIT ligand protein, which has a vital function in the pigment formation process (Yang et al., 2018). The research of *KITLG* has made some achievements in terms of mammalian coat color. The hair color of humans living in Europe is impacted by the regulatory region and variant of *KITLG* (Sulem et al., 2007; Guenther et al., 2014). A selection signature study in indigenous Chongming white goats indicated that *KITLG* was strongly selected and is also crucial for pigment intensity in dogs (Gao et al., 2020; Weich et al., 2020). A previous study indicated that several ROHs (Runs of homozygosity, ROH) exist in the *KITLG* region in horses (Metzger et al., 2015). A genome-wide study of six donkey breeds in China revealed *KITLG* as a candidate gene for donkey color, and the formation mechanism of diluted gray pigmentation (Dun phenotype) in wild ass has been reported (Wang et al., 2020; Zhou et al., 2020); however, there are few studies on the coat color of Asiatic wild ass.

The domestication of donkeys promoted early human communication, trade and transportation. Domestic donkeys are a significant transport and economic animal in China, and they are concentrated between 32° and 42° north latitude. Domestic donkey lives in dry and warm climates in northwest, north and southwest China (Chang, 2011). The ancestors of modern domestic donkeys are considered to be Nubian wild ass (*Equus africanus africanus*) and Somali wild ass (*Equus africanus somaliensis*) (Rosenbom et al., 2015), which are subspecies of African wild ass. Mongolian kulan (*Equus hemionus hemionus*) is a kind of subspecies of Asiatic wild ass (Oakenfull et al., 2000), and it is distributed in central and western Asia (Richard et al., 2001). The Gobi regions in Inner Mongolia, Gansu and Xinjiang of northern China constitute the most important remaining stronghold of the Mongolian kulan (Gerritsmann et al., 2016). Kulan is a representative species in deserts (Zhang et al., 2020). They live in harsh natural conditions, can adapt to dry environments nicely, and have the habit of seasonal short-distance migration and cluster activities. Xinjiang donkey is an excellent miniature local breed in China and is mainly distributed in Hotan, Kashgar and Aksu of the Xinjiang Uygur Autonomous Region (Lei et al., 2005). Mongolian Kulan has an earthy yellow coat on its back and a white belly, which is typical of its appearance, but Xinjiang donkey has a mainly gray coat. Interestingly, the hybrid populations of Mongolian kulan male parents and Xinjiang donkey female parents showed highly similar coat color characteristics to kulan, and this color was highly consistent within Kulan hybrid population (Supplementary Figure S1). There is not precedent for introducing the wild donkey lineage to improve domestic donkeys. In addition, it would be of interesting to assess if Kulan hybrids could produce better meat and skin products than the domestic breeds currently being exploited in China. Therefore, an initial genetic characterization of the Kulan hybrid ass population, as the one here presented, can provide a reference for breeding strategies in this species.

Whole-genome sequencing is an important method to evaluate species population structure and population patterns and identify genome regions related to important economic and environmental adaptation traits. We resequenced the whole genome of Mongolian kulan and 29 Kulan hybrids from Qinghe, Altay region, Xinjiang. The results are expected to fully describe the genomic diversity and population structure of this hybrid population and reveal possible signs of natural or artificial selection. For a meaningful exploration of the formation mechanism of hybrid coat color and other traits, it is necessary to conduct in-depth research.

## Materials and Methods

### Sample Collection and Sequencing

We sampled one Mongolian kulan and 29 Kulan hybrids from the farm of Qinghe Mengyuan Biological Technology Co., LTD. (Qinghe, Altay region, Xinjiang) and extracted genomic DNA from ear tissue by the standard phenol-chloroform protocol (Chen, 1995). Paired-end libraries with an average insert size of 50 bp were constructed for each individual, with an average read length of 150 bp. Whole genome re-sequencing was performed by Illumina HiSeq2000 at Novogene Bioinformatics Institute, Beijing, China.

To determine the ancestry proportion of the Kulan hybrids and their genetic differences to other donkeys (Supplementary Table S1), we chose an additional 28 individuals, including Guanzhong donkey (n = 10), Xinjiang donkey (n = 6), Equus africanus (n = 8), and Asiatic wild ass (n = 4).

### Alignments and Variant Identification

After performing quality controls, the cleaned reads were aligned against the *Equus asinus* reference assembly EquAsi1.0 (https://ftp.ncbi.nlm.nih.gov/genomes/all/GCF/016/077/325/GCF_016077325.2_ASM1607732v2/GCF_016077325.2_ASM1607732v2_genomic.fna.gz) using BWA-MEN (Li and Durbin, 2009) with default settings. Duplicate reads were filtered by Picard tools (http://broadinstitute.github.io/picard), and we used the Genome Analysis Toolkit (GATK, version 3.8) (McKenna et al., 2010) to detect single nucleotide polymorphisms (SNPs). After SNP calling, we used the “VariantFiltration” to discard sequencing and alignment artifacts from the SNPs with the parameters“QD < 2.0, FS > 60.0, MQ < 40.0, MQRankSum < −12.5, ReadPosRankSum < −8.0 and SOR >3.0” and mean sequencing depth of variants (all individuals) “<1/3× and >3×”. Then, the annotation file of the reference genome (https://ftp.ncbi.nlm.nih.gov/genomes/all/GCF/016/077/325/GCF_016077325.2_ASM1607732v2/GCF_016077325.2_ASM1607732v2_genomic.gff.gz) was used to obtain functional annotation of the polymorphic sites in all individuals using SNPeff. All the SNPs were counted by effects (downstream_gene_variant, intron_variant, etc) and genomic region (downstream, exon, intron, ect).

### Phylogenetic and Population Structure Analyses

Neighbor-joining (NJ) tree, PCA, and ADMIXTURE methods were used to account for the genetic relationships between Mongolian Kulan ass and the Mongolian Kulan hybrid populations. An unrooted NJ tree based on the matrix of pairwise genetic distances from the autosomal SNP data of 58 donkeys was constructed by PLINK v1.9 (Purcell et al., 2007) with the parameter (-indep-pair-wise 50 5 0.2) and visualized by MEGA v7.0 (Kumar et al., 2018) and FigTree v1.4.3 (http://tree.bio.ed.ac.uk/software/figtree/). The SNPs data was pruned by PLINK to perform Principal Component Analysis (PCA) and ADMIXTURE analysis. PCA was constructed using the smartPCA program of EIGENSOFT v5.0 (Patterson et al., 2006), and ADMIXTURE v1.3 (Alexander and Lange, 2011) was used for the construction of the population structure.

### Genetic Diversity, Linkage Disequilibrium and Runs of Homozygosity Detection

We calculated the nucleotide diversity for the 58 individuals with 50 kb windows and 50 kb increments using VCFtools (Danecek et al., 2011). PopLDdecay (Zhang C et al., 2019) was used to calculate and visualize the linkage disequilibrium (LD) decay with default parameters. Runs of homozygosity (ROHs) can evaluate the inbreeding degree of the population, and ROHs were identified the --homozyg option implemented in PLINK with the parameter (-homozyg-window-snp 50).

### Genome-wide Selective Sweep Analysis

To detect selective sweeps in the studied Kulan hybrids, we used Guanzhong donkeys as a reference population, as Guangzhong donkeys are typical Chinese domestic donkeys (Shen et al., 2021) and their black coats are highly constant in the Guanzhong population. The four analysis methods used are described as follows. Large differences in genetic diversity (θπ-ratio) were calculated with 50 kb sliding windows and 20 kb steps along the autosomes using VCFtools and in-house scripts. Cross Population Extended Haplotype Homozogysity (XP-EHH) based on the extended haplotype statistics for population pairs was conducted by selscan v1.1 with 50 kb windows (Szpiech and Hernandez, 2014). For the XP-EHH selection scan, our test statistic was the average normalized XP-EHH score in each 50 kb region. The composite likelihood ratio (CLR) uses information from allele frequencies to detect selective scans and relies on determining skews in the allele spectrum to bias rare and frequent alleles, and the CLR test was calculated for sites in non-overlapping 50 kb windows by using SweepFinder2 (Vy and Kim, 2015). The θπ test calculates the nucleotide diversity to explain the base diversity of the genome within the population, and we performed SweeD (Pavlidis et al., 2013) for θπ with 50 kb windows. All the SNPs identified after GATK and variant quality control (excluding those located on chromosomes X, Y and mitochondrial DNA) were used to perform a genome-wide selection sweep analysis with the four analysis methods previously described. The top 0.05% candidate windows identified by the different methods were considered as potential candidate selection sweep regions. The detection of selective clearance is to scan the gene sequence regularly through a fixed scale, and the window is the scale which the software scans the genome at a time. Bedtools (Quinlan and Hall, 2010) was used for the annotation of candidate windows. To gain a better understanding of the biological functions and pathways of the identified candidate genes, Kyoto Encyclopedia of Genes and Genomes (KEGG) pathway analyses was performed using KOBAS 3.0 (Xie et al., 2011). Only FDR (false discovery rate, FDR) corrected *p*-values < 0.05 will be considered and given in the supplementary table.

In the present study, 58 whole genomes of donkeys were analyzed. The potential signatures of positive selection between the Kulan hybrids and Guanzhong donkey were evaluated by θπ-ratio and XP-EHH, and CLR and θπ revealed the selection pressure within the Kulan hybrids.

## Results

### Resequencing of the Kulan Hybrids and Variant Identification

A total of 30 resequenced data datasets, including 1 Mongolian kulan and 29 Kulan hybrids (Supplementary Table S1), were combined with 28 samples from published data (Africa wild ass = 8, Guanzhong donkey = 10, Xinjiang donkey = 6, Asiatic wild ass = 4) (Supplementary Table S2) to form a 58-individual dataset, in which the average depth of the reads was approximately 12.6× coverage. Overall, 6.2 billion clean reads were generated, and the average alignment rate was 99.6% in the 30 individuals (Supplementary Table S1). Using GATK, we detected 23520252 biallelic SNPs in all individuals, and then we annotated them (Supplementary Table S3). The annotated results indicated 209262 SNPs distributed in exons and 7266346 SNPs distributed in introns.

### Phylogenetic Relationship, Principal Component Analysis and Population Structure

**FIGURE 1:**
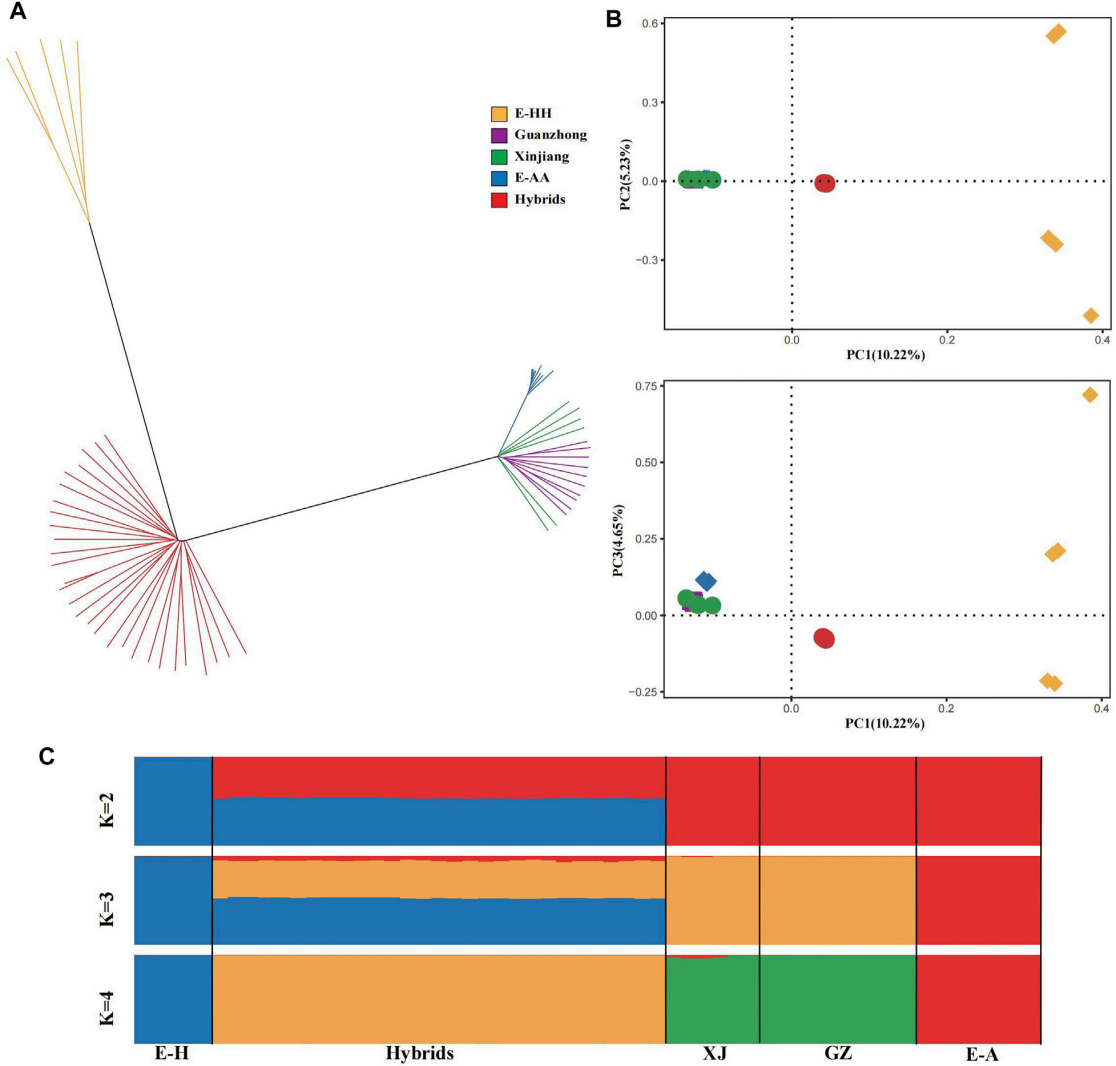
The genetic structure of the donkey populations. **(A)** Neighbor-joining tree of the phylogenetic relationships identified among 58 donkeys analyzed, distributed as 8 E-AA in blue (Africa wild ass, *Equus-asinus-africanus*), 10 Guanzhong in purple (Chinese donkey breed), 6 Xinjiang in green (Chinese donkey breed), 5 E-HH in yellow (Asiatic wild ass, *Equus-hemionus*), 29 Kulan hybrids in red (Mongolian Kulan♂×Xinjiang♀). **(B)** Principal Component Analysis of 5 donkey breeds. **(C)** Bayesian model-based clustering (from K = 2 to K = 4) of 58 donkeys.

Based on the genomic SNPs, we built a neighbor-joining (NJ) tree, principal component analysis and ADMIXTURE (Figure 1). In the NJ tree, all the Kulan hybrids formed a separate branch, and the branch of African wild ass was close to Chinese domestic donkeys, which included Xinjiang and Guanzhong donkeys. In addition, the Asiatic wild ass was far from the others (Figure 1A). The PCA provided similar results (Figure 1B). The first principal component, explaining 10.22% of the total variation, separated Chinese domestic donkeys and African wild ass from Asiatic wild ass and the Kulan hybrids, and the third PC, explaining 4.65% of the total variation, could dissociate Chinese domestic donkeys from African wild ass. In the ADMIXTURE analysis (Figure 1C), when K = 2, the African wild ass lineage and Asiatic wild ass lineage could be distinguished. The Asiatic wild ass, African wild ass and Chinese domestic ass can be distinguished at K = 3 (the lowest CV error, Supplementary Table S4), while the Asiatic wild ass lineage and the Chinese domestic donkey lineage had similar proportions in the Kulan hybrids. There was no doubt that the Kulan hybrids were influenced by the African wild ass, because the African wild ass was the ancestor of the Chinese domestic donkey (Beja-Pereira et al., 2004; Rossel et al., 2008). When K = 4, Asiatic wild ass, African wild ass, Chinese domestic donkey and the Kulan hybrids were independent of each other.

### Patterns of Genomic Variation

**FIGURE 2:**
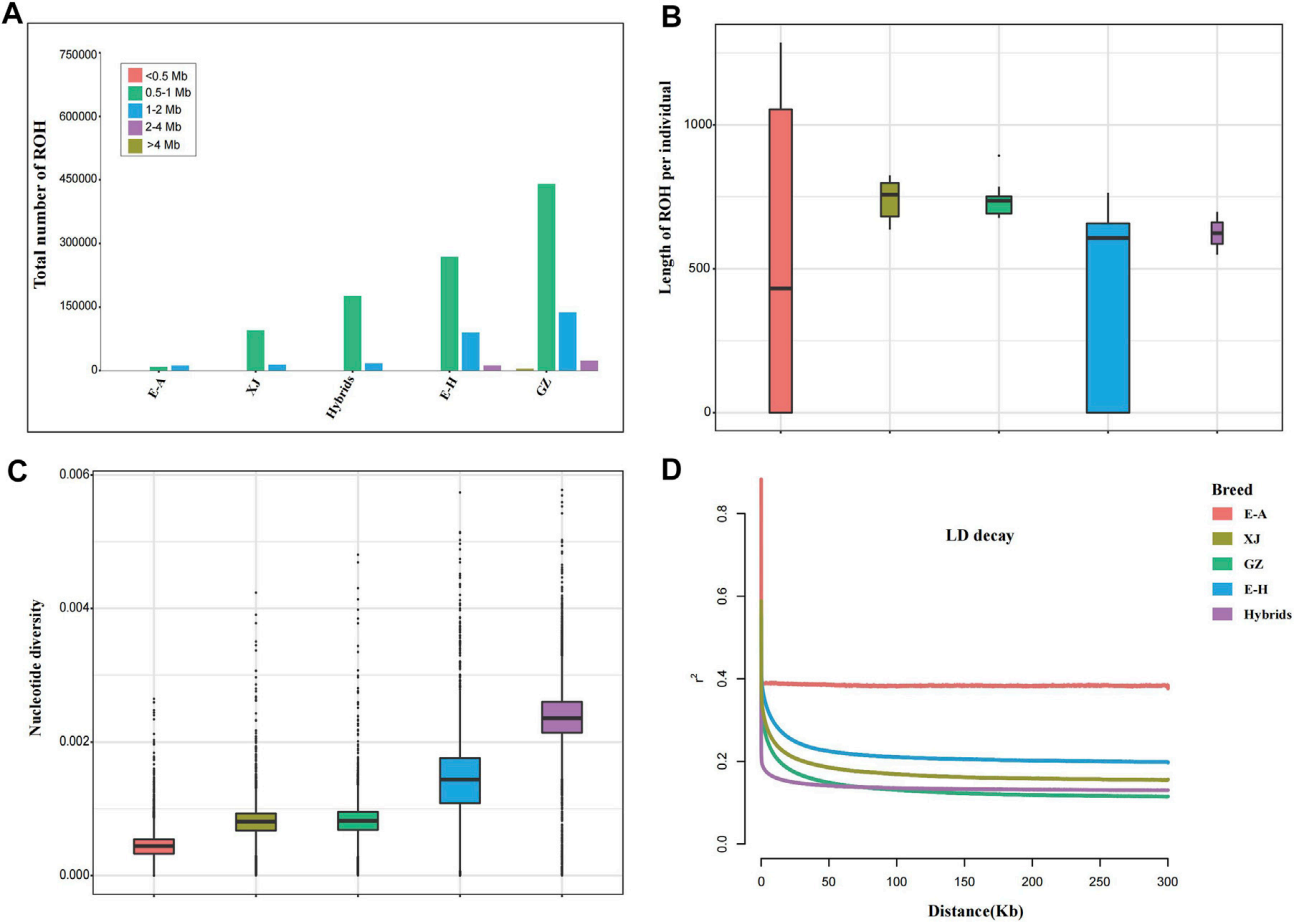
Summary statistics for genomic variation. **(A)** The distribution of the total number of ROHs across chromosomes. **(B)** The distribution of lengths of ROHs in each breed. **(C)** Genome-wide distribution of nucleotide diversity of each breed in 50 kb windows with 50 kb steps. The horizontal line inside the box indicates the median of this distribution; box limits indicate the first and third quartiles; and points show outliers. Data points outside the whiskers can be considered outliers. **(D)** Genome-wide average LD decay estimated from each breed.

Runs of homozygosity (ROHs) can evaluate the inbreeding degree of a population. We identified the length of ROHs into five types: <0.5 Mb, 0.5–1 Mb, 1–2 Mb, 2–4 Mb, and >4 Mb (Figure 2A). Long ROH fragments reflect inbreeding of recent generations, while short ROH fragments indicate inbreeding of distant generations, because the shorter the generation is, the less likely the ROH fragment will be interrupted by recombination. The majority of ROHs we identified in the five breeds were between 0.5–1 Mb. The length of ROHs in African wild ass and the hybrid population was longer than that in the other populations, which suggests that they have inbred in recent generations (Figure 2B). For nucleotide diversity, the Kulan hybrids had the highest value, while the African wild ass had the lowest value (Figure 2C). Linkage disequilibrium decay indicates that the genetic diversity of hybrid populations is high due to artificial hybridization, while African wild ass has the fastest rate of decay (Figure 2D).

### Genome-wide Selective Sweep Test

We applied CLR and θπ methods to detect genomic regions related to selection in the Kulan hybrids. The top 0.05% of windows detected by the two methods were considered candidate regions in the Kulan hybrids. A total of 301 genes were detected by θπ (Supplementary Table S5), and 249 genes were detected by CLR (Supplementary Table S6). After overlapping, 77 genes shared by the two methods were obtained (Supplementary Table S7), which included several significant immune genes (*DEFA1*, *DEFA5*, *DEFA7*, *GIMAP4*, *GIMAP1*, *IGLC1*, and *IGLL5*). Numerous studies have shown that these genes play an integral role in the immune response. The hybrid population may have inherited the strong immunity of the Asiatic wild ass against natural pathogens. After enrichment analysis these 77 genes by KOBAS, we obtained 20 significant KEGG pathways when the corrected *p*-value < 0.05 (Supplementary Table S8).

XP-EHH and θπ-ratio are the methods that detect the selective sweep regions between two populations. With Guanzhong donkey as the reference population, a total of 790 (Supplementary Table S9) and 782 genes (Supplementary Table S10) were obtained using θπ-ratio and XP-EHH, respectively, while 346 genes were common (Supplementary Table S11). We performed functional enrichment to the 346 genes using KOBAS (http://kobas.cbi.pku.edu.cn/) to find vital KEGG pathways, and there were 11 pathways in the enrichment results (Supplementary Table S12). “Melanogenesis” pathway is essential for color phenotype in animals, and *KITLG* gene in the pathway has been proved to be associated with the phenotype of horse (Voß et al., 2020), donkey (Wang et al., 2020; Zhou et al., 2020), human (Yang et al., 2018) and dog (Weich et al., 2020).

## Discussion

In ancient China, donkey provided an excellent approach to servitude, but with the improvement of agricultural mechanization, the donkey industry has expanded the production of donkey skin and meat (Bennett and Pfuderer, 2020). This newer use of donkey poses a long-term challenge that has led to poor donkey breeding at present.

Understanding the population structure of Kulan hybrids can help to figure out the relatedness among the hybrids and other donkey breeds. Under the circumstance of knowing the origin of the Kulan hybrids, we built the NJ tree and performed PCA to explore the pinpoint of the Kulan hybrids at the population level. There was a clear distinction between the Kulan hybrids and the two wild breeds. Furthermore, the Chinese domestic donkey originated from the African wild ass (Beja-Pereira et al., 2004; Rossel et al., 2008), which caused those individuals to gather in one branch. The PCA was consistent with the NJ tree, and they both explained the specificality of the Kulan hybrids. The regular ADMIXTURE revealed the ancestral contributions of the Kulan hybrids, and the Asiatic wild ass contributed almost equally to the Kulan hybrids as the Chinese domestic donkey. Remarkably, a small portion of African wild ass SNPs existed in the Kulan hybrids at K = 3 (the lowest CV error), which agreed with the origin of the Chinese domestic donkey. When K = 4, the existence of the African wild ass lineage also revealed the uneven distribution of Xinjiang donkey in the NJ tree.

The length of ROH fragments can infer the history of inbreeding. Long ROH fragments reflect inbreeding in recent generations, while short ROH fragments indicate inbreeding in distant generations, because the shorter the generation is, the lower the possibility that the ROH fragments are interrupted by recombination (Ceballos et al., 2018). Because of the small number of wild donkeys in this study and the great differences among wild donkey subspecies, the results in Figure 2B are not satisfactory. Nucleotide diversity is used to describe the strength of polymorphisms in a population (Nei and Li, 1979). The Kulan hybrids had the highest nucleotide diversity and this was significantly higher than other populations with strong linkage to hybridization events. In addition, the patterns of LD were consistent with the nucleotide diversity results.

In the process of natural selection, positive selection usually results in a decrease in genetic polymorphism of the selected sites, while the accumulation of favorable mutations often leads to a hitchhiking effort or a selective sweep (Satta et al., 2018). Here, we used four methods to detect selective sweep regions, including θπ, CLR, XP-EHH and θπ-ratio.

**FIGURE 3:**
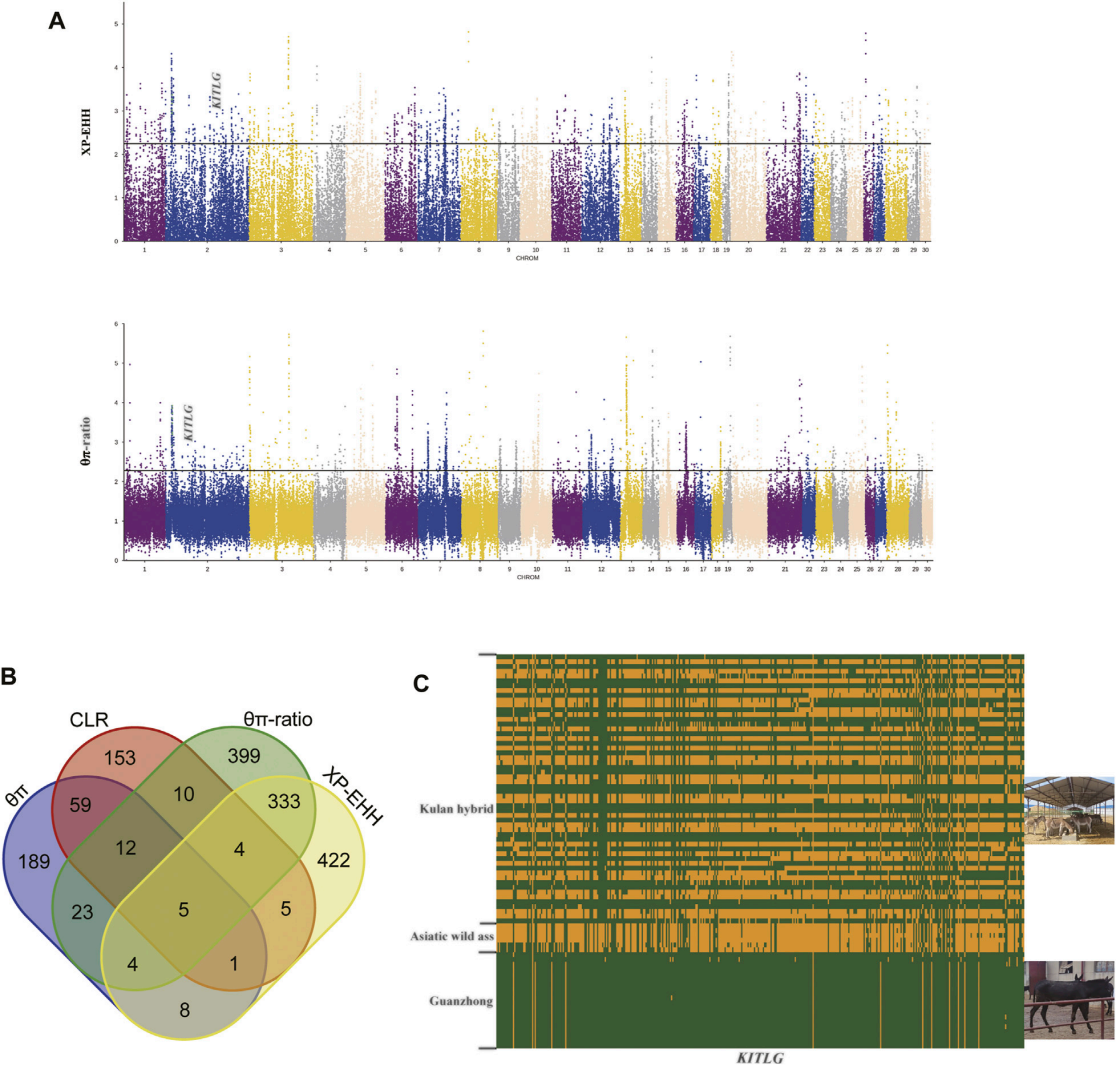
Analysis of the signatures of positive selection in the genome. **(A)** Manhattan plot of selective sweeps. **(B)** Venn diagram showing the genes overlapping among θπ, CLR, θπ-ratio and XP-EHH. **(C)** Linkage disequilibrium analysis of the SNPs in *KITLG* regions.

To determine selective sweeps within the hybrid population, CLR and θπ methods were applied, and we observed 77 overlapping genes with both methods (Figure 3B). We focused on the function of the 77 genes, and some of them belong to the same family. Defensins are a family of antimicrobial and cytotoxic peptides thought to be involved in host defense (Holly et al., 2017), and we obtained three defensin genes (*DEFA1*, *DEFA5*, and *DEFA7*) that had strong signals of selection. In addition, previous studies have shown that *DEFA1* is crucial for the immunity of horses (Bruhn et al., 2009; Schlusselhuber et al., 2012). *GIMAP4* and *GIMAP1* belong to the GTP-binding superfamily and to the immunoassociated nucleotide (IAN) subfamily of nucleotide-binding proteins, which are most highly expressed in immune system cells and involved in T/B-cell development and survival (Heinonen et al., 2015; Saunders et al., 2010). *IGLC1* and *IGLL5* focused on the transcription of immunoglobulins, which are essential for antigen-specific binding in the process of immunity. For the results of KEGG enrichment analysis, most of these pathways are related to immunity, and we want to highlight *HLA-C*, *HLA-A* and *GZMB*, because these genes were obtained in several immune-related KEGG pathways (“Type I diabetes mellitus”, “Allograft rejection”, “Graft-versus-host disease”, “Autoimmune thyroid disease”, “Natural killer cell mediated cytotoxicity”). Human Leukocyte Antigen A (HLA) is also called Major Histocompatibility Complex (HMC), which has been proved to be involved in disease resistance in livestock (Minke et al., 2004; Mikko et al., 1999). *GZMB* encodes a member of the granzyme subfamily of proteins and part of the peptidase S1 family of serine proteases (Turner et al., 2019), and *GZMB* was identified by the four methods simultaneously. *GZMB* is associated with human autoimmune diseases (Xu et al., 2018; Jeong et al., 2021) and essential effector molecules for natural killer (NK)-cell cytotoxicity (Kim et al., 2011). The resistance of cattle to nematode infection and innate immunity requires the participation of *GZMB* (Van Meulder et al., 2013). In xenotransplantation, *GZMB* is often regarded as an important research object (Rodríguez-Gago et al., 2001; Matter-Reissmann et al., 2002). These studies can properly support the key role of *GZMB* in immunity, so the level of immunity of the hybrid population deserves further investigation. Asiatic wild ass has lived in southern Mongolia and northern China for a long time, and these areas have a harsh climate and natural environment. Without good artificial care, Asiatic wild asses struggle with various diseases and emergencies. There is a broad consensus that heterosis is an important method of animal genetic improvement, so Kulan hybrids may inherit strong immunity through hybridization. As a significant economic trait of livestock, disease resistance is considered the focus of breeding efforts. Therefore, wild ass may be a crucial genetic resource for improving the disease resistance of domestic donkeys.

Because the Kulan hybrids that come from Mongolian kulan and Chinese domestic donkeys, we chose Guanzhong donkey as the reference population, which is the representative breed of Chinese domestic donkeys (Shen et al., 2021). To understand the genetic differences between the Kulan hybrids and Guanzhong donkey, θπ-ratio and XP-EHH were performed to find the genomic traces left by selection. We performed enrichment analysis to the 346 overlapping genes (Supplementary Table S11), then we obtained 11 KEGG pathways when corrected *p*-values < 0.05(Supplementary Table S12). In the results, we would like to highlighted several vital terms identified by the analysis, which were “Calcium signaling pathway”, “Regulation of actin cytoskeleton”, “Melanogenesis”. Among these terms, in relation to the phenotypes for which the two groups of animals compared in our pair-wise analyses differ, the Kulan hybrids and Guanzhong donkey, we would highlight the “Melanogenesis” pathway term (*WNT2B*, *GNAO1*, *KITLG*, *Camk2a*, *WNT6*, *DVL3*). Coat color is the most visualized feature of Asiatic wild ass and its Kulan hybrids. The coat color of the Kulan hybrids is earthy yellow while the Guanzhong donkey is black. Compared with Guanzhong donkey, *KITLG* was found in the top 0.5% of regions identified by both methods, then we built a heatmap of haplotypes to verify its true distribution. It was obvious that the Kulan hybrids and other populations had different patterns (Figure 3C). The *KITLG* gene is located on chromosome 2 of donkey, and *KITLG* have been proven to be involved in the formation of coat color in several species (Anello et al., 2019; Voß et al., 2020; Weich et al., 2020; Wen et al., 2021; Wu et al., 2019; Yang et al., 2018). Furthermore, there have been some studies on donkey and horse (Voß et al., 2020; Zhou et al., 2020). Therefore, our findings may provide new insights into the coat color formation of Asiatic wild ass and its Kulan hybrids. Besides, the “calcium signaling pathway” (*PDGFRB*, *Atp2b2*, *ATP2A3*, *PDE1B*, *Lap3*, *MYLK2*, *Camk2a*, *P2RX1*, *ATP2B2*, *P2RX5*) and “Regulation of actin cytoskeleton” (*PDGFRB*, *NCKAP1L*, *SOS1*, *MOS*, *Arhgef7*, *Lap3*, *MYLK2*, *ITGA5*, *ITGAE*) were also identified as the relevant terms based on the results of the KEGG enrichment analyses. Interestingly, the “calcium signalling pathway” and“Regulation of actin cytoskeleton” were related to meat quality (Cao et al., 2019; Malila et al., 2020; Xia et al., 2021) and from the mentioned genes we would highlight the association previously reported for *PDE1B* and *MYLK2.* Haplotypes based on the SNPs derived from a bacterial artificial chromosome containing the bovine gene *PDE1B* were associated with traits related to carcass fat (Stone et al., 2005) and the quantitative trait loci for fat deposition and carcass traits have been identified in the vicinity of the gene encoding *PDE1B* (Shin et al., 2012). *MYLK2* gene was associated with myosin components and may influence muscle development of early porcine embryos through miRNA regulatory network (Zhang X et al., 2019). Heterosis is common in animal breeding, and the Kulan hybrids may be able to provide higher quality meat than the Guanzhong donkey in point of genome-wide selective sweep test, but further research is needed to confirm it. Because of the large wide range of analyses presented here to assess the genetic diversity of the considered populations, and considering that this is not a deep study of selection sweep mapping, the results of the selection sweep mapping regions were assessed only through the enrichment analyses that have been discussed. However, it is important to clarify that some of the selection sweeps may be related to target selection genes that have not been highlighted by the global enrichment analyses. This is the case of the *LCORL* gene which was identified by both, the θπ-ratio and XP-EHH methods, as a candidate gene for a selection sweep located on chromosome 3. This gene is known gene to regulate the body size of livestock species (Makvandi-Nejad et al., 2012; Takasuga, 2016), and previous studies have clearly shown the association between *LCORL* and donkey body size (Shen et al., 2021). Xinjiang donkey is a typical small donkey while Guanzhong donkey is a large breed, and the body size of Kulan hybrids is similar with Xinjiang donkey. Therefore, the identification of this selection sweeps confirms previous studies on the association of *LCORL* and body size. In any case, as previously said, further deeper analyses could be done on the selection sweeps reported here with the objective of identifying potential causal gene and mutations that could directly explain the selection signals here described.

Mongolian Kulan is an essential part of Asiatic wild ass, but hunting and deteriorating living conditions have caused their numbers to plummet leading them to nearly the level of a threatened species in the International Union for Conservation of Nature Red list. As donkey genetic resources are being exhausted, breeding planning would help improve and conserve Chinese native donkeys. For this purpose, our results provide a basis for exploring the genomic characteristics of Asiatic wild ass and its Kulan hybrids in relation to crucial economic traits.

## Conclusion

Based on whole genome re-sequencing data, we provide an overview of Asiatic wild ass and its hybrids. Population genetic structure and genomic diversity analysis identified a new direction for the genetic improvement of Chinese domestic donkeys. Furthermore, we identified genes related to immunity, coat color and meat quality in the Kulan hybrids and the results may serve as valuable resources for research into disease-resistance breeding and the mechanism of animal coat color formation.

## Data Availability

The datasets presented in this study can be found in online repositories. The names of the repository/repositories and accession number(s) can be found in the article/Supplementary Material.

## References

[B1] Alexander D. H. Lange K. (2011). Enhancements to the ADMIXTURE Algorithm for Individual Ancestry Estimation. BMC Bioinforma. 12, 246. 10.1186/1471-2105-12-246 PMC3146885 21682921

[B2] Anello M. Daverio M. S. Silbestro M. B. Vidal‐Rioja L. Di Rocco F. (2019). Characterization and Expression Analysis of KIT and MITF ‐M Genes in Llamas and Their Relation to White Coat Color. Anim. Genet. 50 (2), 143–149. 10.1111/age.12769 30730042

[B3] Beja-Pereira A. England P. R. Ferrand N. Jordan S. Bakhiet A. O. Abdalla M. A. (2004). African Origins of the Domestic Donkey. Science 304 (5678), 1781. 10.1126/science.1096008 15205528

[B4] Bennett R. Pfuderer S. (2020). The Potential for New Donkey Farming Systems to Supply the Growing Demand for Hides. Animals 10 (4), 718. 10.3390/ani10040718 PMC7222848 32326062

[B5] Bruhn O. Paul S. Tetens J. Thaller G. (2009). The Repertoire of Equine Intestinal α-defensins. BMC Genomics 10, 631. 10.1186/1471-2164-10-631 20030839 PMC2803202

[B6] Cao X.-K. Cheng J. Huang Y.-Z. Wang X.-G. Ma Y.-L. Peng S.-J. (2019). Growth Performance and Meat Quality Evaluations in Three-Way Cross Cattle Developed for the Tibetan Plateau and Their Molecular Understanding by Integrative Omics Analysis. J. Agric. Food Chem. 67 (1), 541–550. 10.1021/acs.jafc.8b05477 30596412

[B7] Ceballos F. C. Joshi P. K. Clark D. W. Ramsay M. Wilson J. F. (2018). Runs of Homozygosity: Windows into Population History and Trait Architecture. Nat. Rev. Genet. 19 (4), 220–234. 10.1038/nrg.2017.109 29335644

[B8] Chang H. (2011). Animal Genetic Resources in China-Horses, Donkeys, and Camels. Beijing: China Agricultural Press. Reprinted.

[B66] Chen H. Leibenguth F. (1995). Studies on Multilocus Fingerprints, RAPD Markers, and Mitochondrial DNA of a Gynogenetic Fish (Carassius Auratus Gibelio). Biochem. Genet. 33, 297–306. 10.1007/BF02399929 8748455

[B9] Danecek P. Auton A. Abecasis G. Albers C. A. Banks E. DePristo M. A. (2011). The Variant Call Format and VCFtools. Bioinformatics 27 (15), 2156–2158. 10.1093/bioinformatics/btr330 21653522 PMC3137218

[B10] Gao J. Lyu Y. Zhang D. Reddi K. K. Sun F. Yi J. (2020). Genomic Characteristics and Selection Signatures in Indigenous Chongming White Goat (*Capra hircus*). Front. Genet. 11, 901. 10.3389/fgene.2020.00901 32973871 PMC7472782

[B11] Gerritsmann H. Stalder G. L. Kaczensky P. Buuveibaatar B. Payne J. Boldbaatar S. (2016). Arterial pH and Blood Lactate Levels of Anesthetized Mongolian Khulan (*Equus hemionus* Hemionus) in the Mongolian Gobi Correlate with Induction Time. J. Wildl. Dis. 52, 642–646. Reprinted. 10.7589/2015-07-198 27243152

[B12] Guenther C. A. Tasic B. Luo L. Bedell M. A. Kingsley D. M. (2014). A Molecular Basis for Classic Blond Hair Color in Europeans. Nat. Genet. 46 (7), 748–752. 10.1038/ng.2991 24880339 PMC4704868

[B13] Heinonen M. T. Kanduri K. Lähdesmäki H. J. Lahesmaa R. Henttinen T. A. (2015). Tubulin‐ and Actin‐associating GIMAP4 Is Required for IFN‐γ Secretion during Th Cell Differentiation. Immunol. Cell Biol. 93 (2), 158–166. 10.1038/icb.2014.86 25287446 PMC4355353

[B14] Holly M. K. Diaz K. Smith J. G. (2017). Defensins in Viral Infection and Pathogenesis. Annu. Rev. Virol. 4 (1), 369–391. 10.1146/annurev-virology-101416-041734 28715972

[B15] Jeong K.-H. Kim S. K. Seo J.-K. Shin M. K. Lee M.-H. (2021). Association of GZMB Polymorphisms and Susceptibility to Non-segmental Vitiligo in a Korean Population. Sci. Rep. 11 (1), 397. 10.1038/s41598-020-79705-0 33431938 PMC7801456

[B65] Kim T. Lee S. U. Yun S. Sun H. Lee S. H. Kim J. W. (2011). Human MicroRNA-27a* Targets Prf1 and GzmB Expression to Regulate NK-Cell Cytotoxicity. Blood 118, 5476–5486. 10.1182/blood-2011-04-347526 21960590 PMC3217350

[B16] Klungland H. Våge D. I. (2003). Pigmentary Switches in Domestic Animal Species. Ann. Ny. Acad. Sci. 994, 331–338. 10.1111/j.1749-6632.2003.tb03197.x 12851333

[B17] Kumar S. Stecher G. Li M. Knyaz C. Tamura K. (2018). MEGA X: Molecular Evolutionary Genetics Analysis across Computing Platforms. Mol. Biol. Evol. 35, 1547–1549. Reprinted. 10.1093/molbev/msy096 29722887 PMC5967553

[B18] Lei C. Z. Chen H. Yang G. S. Sun W. B. Lei X. Q. Ge Q. L. (2005). Study on Mitochondrial DNA D-Loop Polymorphism in Chinese Donkeys. J Genet Genom 32, 481–486. in chinese http://europepmc.org/abstract/MED/16018258. 16018258

[B19] Li H. Durbin R. (2009). Fast and Accurate Short Read Alignment with Burrows-Wheeler Transform. Bioinformatics 25 (14), 1754–1760. 10.1093/bioinformatics/btp324 19451168 PMC2705234

[B20] Makvandi-Nejad S. Hoffman G. E. Allen J. J. Chu E. Gu E. Chandler A. M. (2012). Four Loci Explain 83% of Size Variation in the Horse. PLoS One 7 (7), e39929. 10.1371/journal.pone.0039929 22808074 PMC3394777

[B21] Malila Y. Uengwetwanit T. Arayamethakorn S. Srimarut Y. Thanatsang K. V. Soglia F. (2020). Transcriptional Profiles of Skeletal Muscle Associated with Increasing Severity of White Striping in Commercial Broilers. Front. Physiol. 11, 580. 10.3389/fphys.2020.00580 32612536 PMC7308426

[B22] Matter-Reissmann U. B. Forte P. Schneider M. K. J. Filgueira L. Groscurth P. Seebach J. D. (2002). Xenogeneic Human NK Cytotoxicity against Porcine Endothelial Cells Is Perforin/granzyme B Dependent and Not Inhibited by Bcl-2 Overexpression. Xenotransplantation 9 (5), 325–337. 10.1034/j.1399-3089.2002.01074.x 12199864

[B23] McKenna A. Hanna M. Banks E. Sivachenko A. Cibulskis K. Kernytsky A. (2010). The Genome Analysis Toolkit: A MapReduce Framework for Analyzing Next-Generation DNA Sequencing Data. Genome Res. 20 (9), 1297–1303. 10.1101/gr.107524.110 20644199 PMC2928508

[B24] Metzger J. Karwath M. Tonda R. Beltran S. Águeda L. Gut M. (2015). Runs of Homozygosity Reveal Signatures of Positive Selection for Reproduction Traits in Breed and Non-breed Horses. BMC Genomics 16, 764. 10.1186/s12864-015-1977-3 26452642 PMC4600213

[B25] Mikko S. Røed K. Schmutz S. Andersson L. (1999). Monomorphism and Polymorphism at Mhc DRB Loci in Domestic and Wild Ruminants. Immunol. Rev. 167, 169–178. 10.1111/j.1600-065x.1999.tb01390.x 10319259

[B26] Minke J. M. Audonnet J.-C. Fischer L. (2004). Equine Viral Vaccines: The Past, Present and Future. Vet. Res. 35 (4), 425–443. 10.1051/vetres:2004019 15236675

[B27] Nei M. Li W. H. (1979). Mathematical Model for Studying Genetic Variation in Terms of Restriction Endonucleases. Proc. Natl. Acad. Sci. U.S.A. 76 (10), 5269–5273. 10.1073/pnas.76.10.5269 291943 PMC413122

[B28] Oakenfull E. N. Lim H. N. Ryder O. A. (2000). A Survey of Equid Mitochondrial DNA: Implications for the Evolution, Genetic Diversity and Conservation of Equus. Conserv. Genet. 1 (4), 341–355. 10.1023/A:1011559200897

[B29] Patterson N. Price A. L. Reich D. (2006). Population Structure and Eigenanalysis. PLoS Genet. 2 (12), e190. 10.1371/journal.pgen.0020190 17194218 PMC1713260

[B30] Pavlidis P. Živković D. Stamatakis A. Alachiotis N. (2013). SweeD: Likelihood-Based Detection of Selective Sweeps in Thousands of Genomes. Mol. Biol. Evol. 30 (9), 2224–2234. 10.1093/molbev/mst112 23777627 PMC3748355

[B31] Purcell S. Neale B. Todd-Brown K. Thomas L. Ferreira M. A. R. Bender D. (2007). PLINK: A Tool Set for Whole-Genome Association and Population-Based Linkage Analyses. Am. J. Hum. Genet. 81 (3), 559–575. 10.1086/519795 17701901 PMC1950838

[B32] Quinlan A. R. Hall I. M. (2010). BEDTools: A Flexible Suite of Utilities for Comparing Genomic Features. Bioinformatics 26, 841–842. Reprinted. 10.1093/bioinformatics/btq033 20110278 PMC2832824

[B34] Richard P. R. Henry M. M. Badamjaviin L. Claudia F. David P. K. Dulamtseren S. (2001). Status and Distribution of Khulan ( *Equus hemionus* ) in Mongolia. J. Zool. 254 (3), 381–389. 10.1017/S0952836901000887

[B36] Rodríguez-Gago M. de Heredia A. Ramírez P. Parrilla P. Aparicio P. Yélamos J. (2001). Human Anti-porcine Gammadelta T-Cell Xenoreactivity Is Inhibited by Human FasL Expression on Porcine Endothelial Cells. Transplantation 72 (3), 503–509. 10.1097/00007890-200108150-00024 11502983

[B37] Rosenbom S. Costa V. Al-Araimi N. Kefena E. Abdel-Moneim A. S. Abdalla M. A. (2015). Genetic Diversity of Donkey Populations from the Putative Centers of Domestication. Anim. Genet. 46 (1), 30–36. 10.1111/age.12256 25516010

[B38] Rossel S. Marshall F. Peters J. Pilgram T. Adams M. D. O'Connor D. (2008). Domestication of the Donkey: Timing, Processes, and Indicators. Proc. Natl. Acad. Sci. U.S.A. 105 (10), 3715–3720. 10.1073/pnas.0709692105 18332433 PMC2268817

[B39] Satta Y. Fujito N. T. Takahata N. (2018). Nonequilibrium Neutral Theory for Hitchhikers. Mol. Biol. Evol. 35 (6), 1362–1365. 10.1093/molbev/msy093 29722819

[B40] Saunders A. Webb L. M. C. Janas M. L. Hutchings A. Pascall J. Carter C. (2010). Putative GTPase GIMAP1 Is Critical for the Development of Mature B and T Lymphocytes. Blood 115 (16), 3249–3257. 10.1182/blood-2009-08-237586 20194894

[B41] Schlusselhuber M. Jung S. Bruhn O. Goux D. Leippe M. Leclercq R. (2012). *In Vitro* Potential of Equine DEFA1 and eCATH1 as Alternative Antimicrobial Drugs in Rhodococcosis Treatment. Antimicrob. Agents Chemother. 56 (4), 1749–1755. 10.1128/AAC.05797-11 22232283 PMC3318344

[B42] Shen J. Yu J. Dai X. Li M. Wang G. Chen N. (2021). Genomic Analyses Reveal Distinct Genetic Architectures and Selective Pressures in Chinese Donkeys. J. Genet. Genomics 48 (8), 737–745. 10.1016/j.jgg.2021.05.012 34373218

[B43] Shin S. Heo J. Yeo J. Lee C. Chung E. (2012). Genetic Association of Phosphodiesterase 1B (PDE1B) with Carcass Traits in Korean Cattle. Mol. Biol. Rep. 39 (4), 4869–4874. 10.1007/s11033-011-1280-6 21960012

[B44] Stone R. T. Casas E. Smith T. P. L. Keele J. W. Harhay G. Bennett G. L. (2005). Identification of Genetic Markers for Fat Deposition and Meat Tenderness on Bovine Chromosome 5: Development of a Low-Density Single Nucleotide Polymorphism Map. J. Anim. Sci. 83, 2280–2288. Reprinted. 10.2527/2005.83102280x 16160037

[B45] Sulem P. Gudbjartsson D. F. Stacey S. N. Helgason A. Rafnar T. Magnusson K. P. (2007). Genetic Determinants of Hair, Eye and Skin Pigmentation in Europeans. Nat. Genet. 39 (12), 1443–1452. 10.1038/ng.2007.13 17952075

[B46] Szpiech Z. A. Hernandez R. D. (2014). Selscan: An Efficient Multithreaded Program to Perform EHH-Based Scans for Positive Selection. Mol. Biol. Evol. 31, 2824–2827. Reprinted. 10.1093/molbev/msu211 25015648 PMC4166924

[B47] Takasuga A. (2016). PLAG1 and NCAPG‐LCORL in Livestock. Anim. Sci. J. 87 (2), 159–167. 10.1111/asj.12417 26260584 PMC5042058

[B48] Turner C. T. Lim D. Granville D. J. (2019). Granzyme B in Skin Inflammation and Disease. Matrix Biol. 75-76, 126–140. 10.1016/j.matbio.2017.12.005 29247692

[B49] Van Meulder F. Van Coppernolle S. Borloo J. Rinaldi M. Li R. W. Chiers K. (2013). Granule Exocytosis of Granulysin and Granzyme B as a Potential Key Mechanism in Vaccine-Induced Immunity in Cattle against the Nematode Ostertagia Ostertagi. Infect. Immun. 81 (5), 1798–1809. 10.1128/IAI.01298-12 23478322 PMC3648011

[B50] Voß K. Tetens J. Thaller G. Becker D. (2020). Coat Color Roan Shows Association with KIT Variants and No Evidence of Lethality in Icelandic Horses. Genes 11 (6), 680. 10.3390/genes11060680 PMC7348759 32580410

[B51] Vy H. M. T. Kim Y. (2015). A Composite-Likelihood Method for Detecting Incomplete Selective Sweep from Population Genomic Data. Genetics 200 (2), 633–649. 10.1534/genetics.115.175380 25911658 PMC4492385

[B52] Wang C. Li H. Guo Y. Huang J. Sun Y. Min J. (2020). Donkey Genomes Provide New Insights into Domestication and Selection for Coat Color. Nat. Commun. 11 (1), 6014. 10.1038/s41467-020-19813-7 33293529 PMC7723042

[B53] Weich K. Affolter V. York D. Rebhun R. Grahn R. Kallenberg A. (2020). Pigment Intensity in Dogs Is Associated with a Copy Number Variant Upstream of KITLG. Genes 11 (1), 75. 10.3390/genes11010075 PMC7017362 31936656

[B54] Wen J. Shao P. Chen Y. Wang L. Lv X. Yang W. (2021). Genomic Scan Revealed KIT Gene Underlying White/gray Plumage Color in Chinese Domestic Geese. Anim. Genet. 52 (3), 356–360. 10.1111/age.13050 33644907

[B55] Wu Z. Deng Z. Huang M. Hou Y. Zhang H. Chen H. (2019). Whole-Genome Resequencing Identifies KIT New Alleles that Affect Coat Color Phenotypes in Pigs. Front. Genet. 10, 218. 10.3389/fgene.2019.00218 30949195 PMC6436083

[B56] Xia X. Zhang S. Zhang H. Zhang Z. Chen N. Li Z. (2021). Assessing Genomic Diversity and Signatures of Selection in Jiaxian Red Cattle Using Whole-Genome Sequencing Data. BMC Genomics 22 (1), 43. 10.1186/s12864-020-07340-0 33421990 PMC7796570

[B57] Xie C. Mao X. Huang J. Ding Y. Wu J. Dong S. (2011). KOBAS 2.0: A Web Server for Annotation and Identification of Enriched Pathways and Diseases. Nucleic Acids Res. 39 (Web Server issue), W316–W322. 10.1093/nar/gkr483 21715386 PMC3125809

[B58] Xu M. Liu Y. Liu Y. Li X. Chen G. Dong W. (2018). Genetic Polymorphisms of GZMB and Vitiligo: A Genetic Association Study Based on Chinese Han Population. Sci. Rep. 8 (1), 13001. 10.1038/s41598-018-31233-8 30158536 PMC6115438

[B59] Yang Z. Shi H. Ma P. Zhao S. Kong Q. Bian T. (2018). Darwinian Positive Selection on the Pleiotropic Effects of KITLG Explain Skin Pigmentation and Winter Temperature Adaptation in Eurasians. Mol. Biol. Evol. 35 (9), 2272–2283. 10.1093/molbev/msy136 29961894

[B60] Zhang C. Dong S.-S. Xu J.-Y. He W.-M. Yang T.-L. (2019). PopLDdecay: A Fast and Effective Tool for Linkage Disequilibrium Decay Analysis Based on Variant Call Format Files. Bioinforma. Oxf. Engl. 35 (10), 1786–1788. 10.1093/bioinformatics/bty875 30321304

[B61] Zhang J. Liu F. Cao J. Liu X. (2015). Skin Transcriptome Profiles Associated with Skin Color in Chickens. PLoS One 10 (6), e0127301. 10.1371/journal.pone.0127301 26030885 PMC4452617

[B62] Zhang X. C. Shao C. L. Ge Y. Chen C. Xu W. X. Yang W. K. (2020). Suitable Summer Habitat of the Khulan in the Mt. Kalamaili Ungulate Nature Reserve and Estimation of its Population. Ying Yong Sheng Tai Xue Bao 31 (9), 2993–3004. 10.13287/j.1001-9332.202009.032 33345500

[B63] Zhang X. Cai S. Chen L. Yuan R. Nie Y. Ding S. (2019). Integrated miRNA-mRNA Transcriptomic Analysis Reveals Epigenetic-Mediated Embryonic Muscle Growth Differences between Wuzhishan and Landrace Pigs1. J. Anim. Sci. 97 (5), 1967–1978. 10.1093/jas/skz091 31222274 PMC6488331

[B64] Zhou Z. Fan Y. Wang G. Lai Z. Gao Y. Wu F. (2020). Detection of Selection Signatures Underlying Production and Adaptive Traits Based on Whole-Genome Sequencing of Six Donkey Populations. Animals 10 (10), 1823. 10.3390/ani10101823 PMC7600737 33036357

